# Biparametric versus multiparametric MRI for the detection of clinically significant prostate cancer in a diverse, multiethnic population

**DOI:** 10.1007/s00261-024-04332-6

**Published:** 2024-06-05

**Authors:** Max Abramson, Matthew DeMasi, Denzel Zhu, Laena Hines, Wilson Lin, Devaraju Kanmaniraja, Victoria Chernyak, Ilir Agalliu, Kara L. Watts

**Affiliations:** 1 Albert Einstein College of Medicine, Bronx, NY USA; 2 Department of Urology, University Hospitals Cleveland Medical Center, Cleveland, OH USA; 3 Department of Urology, University of Rochester Medical Center, Rochester, NY USA; 4 Department of Urology, New York University Langone Health, New York, NY USA; 5 Department of Radiology, Memorial Sloan Kettering Cancer Center, New York, NY USA; 6 Department of Radiology, Montefiore Medical Center, Bronx, NY USA; 7 Department of Epidemiology and Population Health, Albert Einstein College of Medicine, Bronx, NY USA; 8 Department of Urology, Montefiore Medical Center, Bronx, NY USA; 9 Department of Urology, Montefiore Medical Center, Albert Einstein College of Medicine, 1250 Waters Place, Tower 1; Penthouse, Bronx, NY 10461 USA

**Keywords:** Prostate cancer, Multiparametric, Biparametric, Magnetic resonance imaging, Prostate biopsy, Multiethnic

## Abstract

**Purpose:**

There is not yet satisfactory performance data comparing multiparametric MRI (mpMRI) versus biparametric MRI (bpMRI) for detecting prostate cancer (PCa), particularly in high-risk populations. We compared both protocols for detecting overall PCa and clinically significant PCa (CS-PCa; defined as Grade Group ≥ 2) in a multiethnic urban population.

**Methods:**

We retrospectively reviewed electronic medical record data from men who underwent image-guided fusion prostate biopsy (FB) between 2016 and 2021 at our institution. Patient characteristics, Prostate Imaging Reporting and Data System (PI-RADS) scores, and FB outcomes were analyzed based on MRI protocol. Multivariate mixed-effects logistic regression models were used to examine associations of bpMRI versus mpMRI for detecting overall PCa and CS-PCa in targeted lesions, among all patients and stratified by race/ethnicity.

**Results:**

Overall, 566 men (44.0% Non-Hispanic Black [NHB]; 27.0% Hispanic) with 975 PI-RADS 3–5 lesions on MRI underwent FB. Of these, 312 (55%) men with 497 lesions underwent mpMRI and 254 (45%) men with 478 lesions underwent bpMRI. On multivariate analyses among all men, the odds of detecting overall PCa (OR = 1.18, 95% CI: 1.05–3.11, p = 0.031) and CS-PCa (OR = 2.15, 95% CI: 1.16–4.00, *p* = 0.014) on FB were higher for lesions identified on bpMRI than mpMRI. When stratified by race/ethnicity, the odds of detecting overall PCa (OR = 1.86; *p* = 0.15) and CS-PCa (OR = 2.20; *p* = 0.06) were not statistically different between lesions detected on bpMRI or mpMRI.

**Conclusion:**

BpMRI has similar diagnostic performance to mpMRI in detecting overall and CS-PCa within a racially/ethnically diverse population. BpMRI can be utilized for evaluating suspected CS-PCa among NHB and Hispanic men.

## Introduction

Prostate cancer (PCa) is the most commonly diagnosed non-skin malignancy and the second most common cause of death amongst men in the United States [1]. Image-guided fusion prostate biopsy (FB) platforms are increasingly being used to detect PCa, with multiple trials showing increased detection rates of clinically significant prostate cancer (CS-PCa) via FB compared to standard, traditional biopsy [2–4]. As FB platforms have been refined, concomitant improvements in magnetic resonance imaging (MRI) technology and techniques for identifying PCa have also been made.

Multiparametric MRI (mpMRI) includes T2-weighted, diffusion-weighted, and dynamic contrast enhanced (DCE) sequences, and is the standard of care for imaging assessment of PCa. In recent years, literature supporting the use of biparametric MRI (bpMRI), which omits DCE sequences, has emerged [5–9]. The most recent version of the Prostate Imaging Reporting and Data System (PI-RADS) v2.1 encourages the use of mpMRI for certain clinical situations. However, the PI-RADS Steering Committee states that there are a lack of data supporting the use of mpMRI over bpMRI as first line imaging and that further comparative studies are needed [6, 10].

Compared to mpMRI, bpMRI has the benefit of reduced examination times, costs, and invasiveness, since no intravenous contrast is needed [6, 7]. Several studies, primarily retrospective and single-center in nature [11], have compared bpMRI to mpMRI and have demonstrated comparable diagnostic performance for the detection of PCa [5, 8–10, 12–16]. However, non-Caucasian patients are vastly underrepresented in the literature and the performance of bpMRI, in particular, has not been well evaluated in high-risk multiethnic populations [17]. Given the aforementioned benefits of bpMRI, we sought to compare the performance of bpMRI versus mpMRI for detecting overall PCa and CS-PCa (as defined by Grade Group ≥ 2) on FB in a diverse, multiethnic urban population.

## Materials and methods

### Study design and patient population

We performed an institutional review board approved, retrospective electronic medical record review of all men who underwent FB for 1 + lesions (PI-RADS ≥ 3) detected on prostate MRI between October 2016 and July 2021 at a large, diverse academic institution. Clinical data including age, race/ethnicity, body mass index (BMI), pre-biopsy prostate specific antigen (PSA), and prior use of 5-alpha reductase inhibitors (5-ARI) were reviewed. MRI data extracted included MRI protocol (bpMRI vs mpMRI), PI-RADS score(s) (3–5) of suspicious lesions, lesion location (peripheral versus transition zone), and prostate volume. Biopsy pathology results for each targeted biopsy (per lesion) and the highest Gleason grade obtained for concomitant systematic biopsy, when performed, were recorded.

### MRI protocol and image analysis

MRI examinations were performed with 1.5T scanners (Ingenia, (Philips Healthcare, Best, Netherlands)) or 3T scanners (Signa Excite HDXt (GEHealthcare, Milwaukee, WI), Ingenia, or Achieva (Philips Healthcare, Best, Netherlands)). Images were acquired with a torso phased-array coil; endorectal coil was not used. The field of view was 18 cm for all relevant sequences. All examinations included axial T2-weighted sequences (slice thickness 3 mm; slice spacing 0 mm; TR 2512–8633 ms; TE 100–113.5 ms; matrix 224–320 × 173–256). Diffusion-weighted sequences were acquired with a maximal b value of 1,000–1,400 s/mm^2^. For DCE-MRI, gadobutrol (Gadavist, Bayer Pharma; 0.05 mmol of gadolinium per kilogram of body weight) was injected at 2 mL/sec followed by a 40-mL saline flush at 2 mL/sec. DCE-MRI was acquired with a temporal resolution of 7–15 s (15–30 phases, slice thickness 4 mm; TR 3.8–5.3 ms; TE 1.7–2.7 ms; matrix 124–144 × 156–192). As of September 2019, DCE-MRI was no longer performed due to our institution switching from mpMRI to bpMRI based on literature available at that time [3, 5, 14]. A select few patients that received bpMRI prior to September 2019 were due to the patient not being able to tolerate or receive IV contrast (e.g. renal failure or contrast allergy).

All examinations were interpreted by one of six abdominal/pelvic radiologists who had completed fellowship training. DynaCAD (Invivo, Philips Healthcare, Best, Netherlands) was used to outline discrete lesions. PI-RADS v2.0 was used to assign a score (1 to 5). A standardized reporting template was used in all cases.

### Biopsy protocol

FBs were performed on men with any prostate MRI lesion (PI-RADS ≥ 3) by one of two academic urologists. All patients received local anesthetic peri-prostatic nerve block via transrectal ultrasound probe. The operator reviewed the MRI images. Ultrasound imaging in both transverse and longitudinal views were obtained. Targeted biopsy of the suspicious lesion(s) was performed (Uronav, Philips Healthcare, Best, Netherlands), followed by a standard 12-core template sampling of the prostate in most patients. Our standard practice is to obtain 3 cores per lesion on targeted biopsy, but there is variation in the number of cores obtained.

### Statistical analysis

Demographic and clinical characteristics for the two cohorts were compared using *χ*^2^-test for categorical variables, Student t-test for independent samples, and Mann–Whitney *U* test/Wilcoxon rank-sum test for continuous normally distributed and non-normally distributed variables, respectively. All tests were 2-sided with a significance level set at *p* < 0.05. Univariate and then multivariate mixed-effects logistic regression models were used to examine associations of bpMRI versus mpMRI and the risk of overall PCa and CS-PCa on the targeted lesions. Multivariate models were adjusted for the following covariates: age, BMI, race/ethnicity, PSA density (PSAD), use of 5-ARI, and PI-RADS score as fixed effect; whereas patients were modelled as a random effect to account for correlations that exist from multiple lesions from the same patient.

Mixed effect logistic regression models were run separately in stratified analyses by race/ethnicity (i.e. Non-Hispanic White (NHW), Non-Hispanic Black (NHB), and Hispanic), by PI-RADS score, by lesion location (peripheral vs transitional zone of the prostate), and by active surveillance (AS) protocol to explore whether the use of bpMRI versus mpMRI in detecting overall PCa and CS-PCa on targeted biopsy was different in strata defined by these covariates. Patients with ‘other’ or missing race/ethnicity were excluded from the stratified analysis by race. All statistical data analyses were performed using Stata v17 (StataCorp, College Station, TX).

## Results

A total of 566 men with 975 PI-RADS ≥ 3 lesions identified on prostate MRI was analyzed. Our cohort comprised 249 (44.0%) NHB men, 103 (18.2%) NHW men, and 153 (27.0%) Hispanic men. Ultimately, 312 (55%) men with 497 lesions identified on mpMRI were compared to 254 (45%) men with 478 lesions identified by bpMRI.

Table 1 compares the characteristics of the two cohorts who underwent mpMRI vs bpMRI. There was a difference in the racial/ethnic distribution between the groups, with a higher proportion of Hispanic men in the bpMRI group, and higher proportion of NHB and NHW men in the mpMRI group. Overall, the target size and lesion location were similar between groups, as were the number of lesions in which PCa and CS-PCa were detected (Table 2). The groups differed with regards to the distribution of PI-RADS lesions: the bpMRI cohort had a higher proportion of PI-RADS 3 lesions (58.4% vs 46.9%, *p* < 0.0001), whereas the mpMRI cohort had a higher proportion of PI-RADS 4 lesions (36.0% vs 24.9%, *p* < 0.0001). Additionally, there was a difference in the number of cores per lesions between the two MRI groups (*p* < 0.001).

**Table 1:** Comparison of demographics and clinical characteristics among all patients and stratified by MRI prostate modality (biparametric (bp) vs multiparametric (mp) MRI)

	All patients	mpMRI	bpMRI	*p*-value
	*N* = 566	*N* = 312	*N* = 254	
Age at biopsy, mean, (SD) (yrs)	63.2 (7.7)	63.5 (7.4)	62.9 (8.0)	0.31
Race/ethnicity, N (%)				**0.01**
Non-Hispanic White	103 (18.2)	63 (20.2)	40 (15.8)	
Non-Hispanic Black	249 (44.0)	146 (46.8)	103 (40.6)	
Hispanic	153 (27.0)	80 (25.6)	73 (28.7)	
Other/Declined	61 (10.8)	23 (7.4)	38 (15.0)	
Insurance, N (%)				**0.01**
Private	210 (37.1)	110 (35.3)	100 (39.4)	
Medicare	217 (38.3)	120 (38.5)	97 (38.2)	
Medicaid	90 (15.9)	44 (14.1)	46 (18.1)	
Other/Uninsured	49 (8.7)	38 (12.2)	11 (4.3)	
BMI, mean, SD (kg/m^2^)	28.7 (5.1)	28.7 (4.9)	28.6 (5.3)	0.97
Pre-operative PSA, median (IQR; ng/mL)	7.3 (5.1–11.1)	7.5 (5.2–11.2)	7.1 (5.0–10.8)	0.41
Prostate volume, median (IQR; mL)	46.6 (33.0–68.4)	48.4 (32.2–70.5)	44.3 (33.9–67.5)	0.75
PSA density, median (IQR; per 10 units of ng/mL^2^)	1.4 (0.9–2.5)	1.4 (0.9–2.5)	1.4 (0.9–2.5)	0.90
5-alpha reductase inhibitor use, N (%)	107 (18.9)	66 (21.2)	41 (16.1)	0.13
Surveillance Biopsy, N (%)	92 (16.3)	53 (17.0)	39 (15.4)	0.60

34 patients that had a 1st biopsy with mpMRI had a 2nd biopsy with bpMRI. The table shows only the data on the 1st biopsy

Bolded *p*-values indicate significance (*p* < 0.05)

**Table 2:** Comparison of bpMRI and mpMRI lesions and lesion outcomes

	All lesions	mpMRI	bpMRI	*p*-value
	*N* = 975	*N* = 497	*N* = 478	
Number of lesions on MRI, N (%)				0.10
One	601 (61.6)	313 (63.0)	288 (60.3)	
Two	271 (27.8)	138 (27.8)	133 (27.8)	
Three	88 (9.0)	35 (7.0)	53 (11.1)	
Four to Six	15 (1.5)	11 (2.2)	4 (0.8)	
Lesion max dimension, median (IQR; cm)	1.3 (0.9–1.8)	1.2 (0.9–1.8)	1.3 (0.9–1.8)	0.94
Number of Cores per Lesion, n (%)				** < 0.001**
1	19 (2.0)	8 (1.6)	11 (2.3)	
2	156 (16.0)	103 (20.7)	53 (11.1)	
3	619 (63.5)	275 (55.3)	344 (72.0)	
4	129 (13.2)	75 (15.1)	54 (11.3)	
5 to 9	19 (2.0)	10 (2.0)	9 (1.9)	
PI-RADS, N (%)				** < 0.01**
3	512 (52.5)	233 (46.9)	279 (58.4)	
4	298 (30.6)	179 (36.0)	119 (24.9)	
5	165 (16.9)	85 (17.1)	80 (16.7)	
Cancer detected in all lesions, N (%)				
Overall PCa	376 (38.6)	179 (36.0)	197 (41.2)	0.10
CS-PCa (GG score ≥ 2)	231 (23.7)	107 (21.6)	124 (25.9)	0.11
Proportions of PI-RADS with overall PCa, N (%)				
PI-RADS 3	109/512 (21.3)	38/233 (16.3)	71/279 (25.5)	**0.01**
PI-RADS 4	152/298 (51.0)	85/179 (47.5)	67/119 (56.3)	0.14
PI-RADS 5	115/165 (69.7)	56/85 (65.9)	59/80 (75.8)	0.27
Proportions of PI-RADS with CS-PCa, N (%)				
PI-RADS 3	52/512 (10.2)	18/233 (7.7)	34/279 (12.2)	0.10
PI-RADS 4	92/298 (30.9)	47/179 (26.3)	45/119 (37.8)	**0.03**
PI-RADS 5	87/164 (53.1)	42/84 (50.0)	45/80 (56.3)	0.42
Location of all lesions, N (%)				0.59
Peripheral zone	655 (67.3)	328 (66.1)	327 (68.6)	
Transitional zone	305 (31.4)	161 (32.5)	144 (30.2)	
Both	12 (1.2)	7 (1.4)	5 (1.1)	

Bolded *p*-values indicate significance (*p* < 0.05)

The rates of overall PCa detected among PI-RADS 3 lesions were higher for lesions identified on bpMRI than mpMRI (25.5% vs 16.3%, *p* = 0.012). Similarly for PI-RADS 4 lesions, there was a higher percentage of CS-PCa detected on targeted biopsy in the bpMRI cohort than mpMRI cohort (37.8% vs 26.3%, *p* = 0.034).

**Fig. 1:**
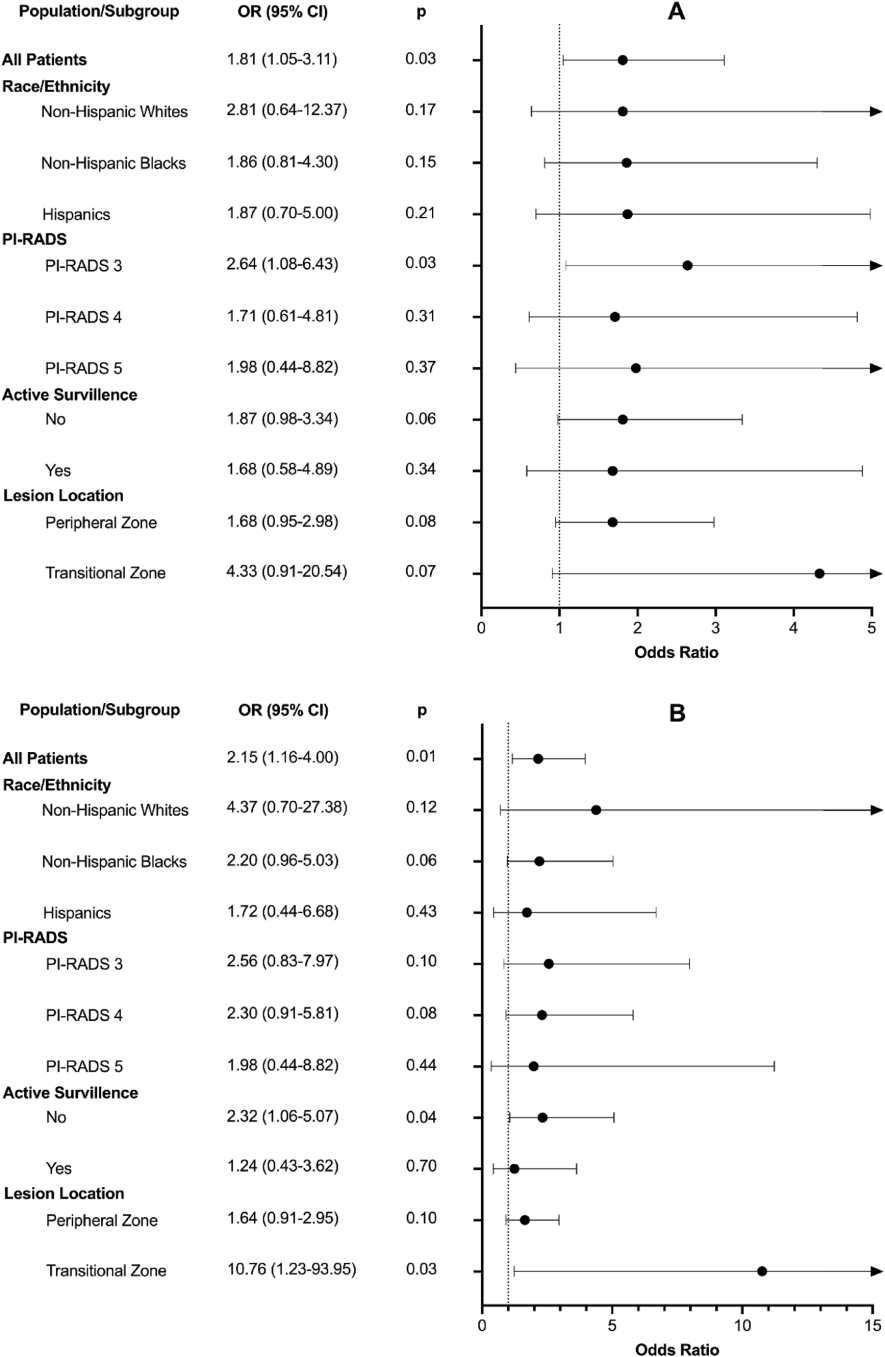
Performance of bpMRI in detecting overall PCa (**A**) and CS-PCa (**B**) in all lesions compared to mpMRI (reference, OR = 1), among all patients and stratified by subgroups. Presented within the tables are the results of a multivariate model comparing the performance of bpMRI to mpMRI (reference, OR = 1). OR less than 1 favor mpMRI, while OR greater than 1 favor bpMRI. The points refer to OR, while the whiskers denote the 95% confidence interval. Arrows denote confidence interval boundaries that were larger than the x-axis. The model has been adjusted for age (years), BMI (kg/m^2^), race/ethnicity (with NHW used as reference), presence of 5-ARI, PSA density (10*ng/mL^2^), and PI-RADS score expressed as a categorical variable (PI-RADS = 3, 4, or 5)

On multivariate analysis, the odds of overall PCa (OR = 1.18, 95% CI: 1.05–3.11, *p* = 0.031) and CS-PCa (OR = 2.15, 95% CI: 1.16–4.00, *p* = 0.014) on targeted biopsy were higher among lesions identified on bpMRI compared to those on mpMRI among all men (Fig. 1). Since the two groups differed by race/ethnicity, we carried out a stratified analysis to compare detection rates of all PCa and CS-PCa among NHB, NHW, and Hispanic patients when analyzing targeted biopsies of lesions identified on bpMRI vs mpMRI. The odds of overall PCa (OR = 1.86; *p* = 0.15) and CS-PCa (OR = 2.20; *p* = 0.06) were not different between lesions detected on bpMRI or mpMRI for NHB men; likewise, the odds were not different between imaging modalities for NHW or Hispanic men.

When stratified by PI-RADS score on multivariate analysis, the yield of overall PCa was higher on targeted biopsy of PI-RADS 3 lesions identified on bpMRI as compared to mpMRI (OR = 2.65, 95% CI: 1.06–6.43, *p* = 0.033). When we examined rates of CS-PCa on targeted biopsy, we did not detect any statistically significant differences in the odds of detecting CS-PCa per PI-RADS lesion stratum. We then performed subgroup analyses comparing patients enrolled in AS protocols and based on lesion location. Among AS patients undergoing surveillance biopsy, we found no difference between bpMRI and mpMRI for detecting overall or CS-PCa. However, among non-AS patients, we found a higher likelihood of detecting CS-PCa with bpMRI (OR = 2.32, 95% CI: 1.06–5.07, *p* = 0.035). Additionally, when examining lesion location, we found bpMRI had a higher likelihood than mpMRI of detecting CS-PCa in lesions within the prostate’s transitional zone (OR = 10.76, 95% CI: 1.23–93.95, *p* = 0.03).

Lastly, we analyzed the number of cores obtained per biopsy lesion between mpMRI and bpMRI protocols. A median of 3 cores (IQR: 3–3) was obtained under both protocol (*p* = 0.17). Similarly, a median of 3 cores was obtained (IQR: 3–3) for all PI-RADS lesions (*p* = 0.18) when stratified by PI-RADS score (3–5).

## Discussion

Our results demonstrate that detection of overall and CS-PCa on FB may be higher in lesions identified on bpMRI compared to those on mpMRI. In various group sub-analyses, bpMRI performed either similarly or somewhat superior to mpMRI. This study adds to the literature supporting bpMRI as an appropriate alternative to mpMRI for PCa evaluation and validates it among a racially diverse patient population [5, 9, 12, 15, 18–20].

We found the odds of detecting overall PCa on FB were higher among PI-RADS 3 lesions using bpMRI than mpMRI, with trends similar but non-significant for CS-PCa. This suggests that PI-RADS 3 lesions detected on bpMRI may require a more aggressive workup, particularly when deciding whether a targeted biopsy is indicated for these typically lower-risk lesions. Similar findings were reported in a study of 123 men that found bpMRI had higher specificity than mpMRI for the detection of CS-PCa [21]. However, in a retrospective review of 235 patients, mpMRI had a higher detection of CS-PCa upon biopsy than bpMRI among patients with PI-RADS 4 lesions [20]. Therefore, larger datasets are needed to better resolve the differences per PI-RADS lesion.

Our analysis of men enrolled in AS protocol had similar outcomes when comparing bpMRI to mpMRI. Similarly, a review of 101 AS patients, who underwent diagnostic FB with bpMRI and follow-up confirmatory MRI with mpMRI, found that confirmatory mpMRI failed to detect higher rates of missed lesions, miscategorized PI-RADS risk assessments, or variation in tumor size, compared to bpMRI alone [22]. Our study supports prior evidence that bpMRI is a sufficient alternative to mpMRI in AS patients, including in high-risk populations.

Our study population was highly diverse, comprising nearly 50% NHB men and nearly 30% Hispanic men. When stratified by race/ethnicity, we found no differences in the odds of yielding PCa or CS-PCa on biopsied lesions identified by either MRI protocol. That said, our results showed a trend towards an increased odds of detecting CS-PCa in NHB men among lesions identified on bpMRI, which approached, but did not reach, significance. A larger study population may be needed to determine if there is a significant difference. Overall, prior work on this topic has been hindered by poor enrollment of racial/ethnic minorities and a lack of reporting of racial/ethnic demographic data. Studies comparing mpMRI to bpMRI in somewhat diverse cohorts have found similar performance for detecting CS-PCa [23–25]. However, NHW men were still vastly overrepresented in these studies, and they did not report sub-analyses stratified by race/ethnicity. Furthermore, a prior study at our institution showed NHB men have higher odds of detecting overall and CS-PCa on FB (both bpMRI and mpMRI) compared to NHW men [26]. Our study adds to the body of evidence that bpMRI is an appropriate diagnostic modality alternative to mpMRI for NHB and Hispanic men.

Our study has several strengths and limitations. Strengths include sample size and enrollment of a diverse patient population. Additionally, we utilized mixed-effects logistic regression, which considers patient-specific and lesion-specific factors when analyzing the odds of detecting PCa on biopsy. Routine multidisciplinary conferences were utilized to review challenging cases, and the urologists and radiologists involved were consistent throughout the study. Limitations include its retrospective nature. Additionally, we did not perform a head-to-head comparison of mpMRI and bpMRI protocols; our patients received either mpMRI or bpMRI based on date ordered (per our institution’s decision to change the imaging protocol). We also did not collect data on which patients received MRI using 1.5T vs 3T instruments. However, studies comparing 1.5T vs 3T instruments have found similar PI-RADS scoring when read by fellowship-trained body radiologists [27, 28].

All exams in this study meet the updated technical parameters recommended in PI-RADS V2.1. Due to logistical challenges in 2020 and the early half of 2021, there was a delay in implementing the imaging interpretation criteria of PI-RADS V2.1 at our institution. All the studies performed during this time were reported using the PI-RADS V2. Recent literature has shown that upgrade and downgrade rates are not significantly different between PI-RADS V2 and V2.1 [29].

Furthermore, our study did not compare the PI-RADS 4 lesions between the 2 groups to differentiate between the dominant PI-RADS 4 lesions on bpMRI and the upgraded PI-RADS 4 lesions on mpMRI that were upgraded by DCE (DWI score 3 with positive DCE). There is varied literature on the benefits of DCE with some studies showing no added benefit to including DCE [9, 13], while other studies have demonstrated benefit only in inexperienced readers [30].

Additionally, our study may be subject to selection bias given that we did not match patients based on patient characteristics between mpMRI and bpMRI protocols. Of note, our patient population had imbalances in the proportion of PI-RADS 3 and 4 lesions between mpMRI and bpMRI protocols. We performed stratified analyses by PI-RADS score to control for this variation, but caution must be applied given the imbalances. Finally, six fellowship-trained body radiologists reviewed our MRI scans, which may contribute to greater inter-reader variability. However, this reflects the reality of clinical practice, and therefore, this study provides results that can be expected in a true clinical setting, as opposed to a strictly controlled research study.

## Conclusion

In conclusion, we found that bpMRI had similar or slightly superior diagnostic performance in the detection of all and CS-PCa compared to mpMRI, within a racially/ethnically diverse patient population. Additionally, among men enrolled in AS, bpMRI had similar performance to mpMRI. Therefore, bpMRI is a safe alternative for the evaluation of PCa among NHB and Hispanic men.

## Data Availability

The data to support this study is not publicly available since it contains potentially identifiable patient information.
